# The quantum-classical complexity of consciousness and orchestrated objective reduction

**DOI:** 10.3389/fnhum.2025.1630906

**Published:** 2025-09-05

**Authors:** Alessandro Sergi, Antonino Messina, Gabriella Martino, Maria Teresa Caccamo, Salvatore Magazù, Giulio Ruffini, Min-Fang Kuo, Michael A. Nitsche

**Affiliations:** ^1^Dipartimento di Scienze Matematiche e Informatiche, Scienze Fisiche e Scienze della Terra, Università degli Studi di Messina, Messina, Italy; ^2^Institute of Systems Science, Durban University of Technology, Durban, South Africa; ^3^Dipartimento di Matematica ed Informatica, Università di Palermo, Palermo, Italy; ^4^Dipartimento di Medicina e Clinica Sperimentale, Università degli Studi di Messina, Messina, Italy; ^5^Neuroelectrics, Barcelona, Spain; ^6^Department of Psychology and Neurosciences, Leibniz Research Centre for Working Environment and Human Factors, TU Dortmund, Dortmund, Germany; ^7^German Center for Mental Health (DZPG), Bochum, Germany; ^8^Protestant Hospital of Bethel Foundation, University Hospital OWL, University Clinic of Psychiatry and Psychotherapy, Bielefeld University, Bielefeld, Germany

**Keywords:** consciousness, neuroscience, microtubules, Orch OR, quantum, quantum-classical dynamics, complexity

## 1 Introduction

The hypothesis that quantum mechanics might underlie the origin of consciousness has a long and intricate history (Jordan, 1932, 1941; Beyler, 1994, 1996; McFadden, 2002; McFadden and Al-Khalili, 2014; Schrödinger, 2013, 2008; Neumann, 1932; Wigner, 1961; London and Bauer, 1939; Penrose, 1989, 1994; Stapp, 1993; Jibu and Yasue, 1995; Hameroff and Penrose, 1996; Penrose and Hameroff, 2017; Ho, 2013; D'Ariano and Faggin, 2002; Georgiev, 2025; Fisher, 2015). In recent years, the theory of Orchestrated Objective Reductions (“Orch OR") (Penrose, 1989, 1994; Hameroff and Penrose, 1996; Hameroff, 2001; Hameroff and Penrose, 2014; Penrose and Hameroff, 2017; Tegmark, 2000; Hagan et al., 2002; McKemmish et al., 2009; Rosa and Faber, 2004) has emerged as one of the main theories for understanding the function of the brain and the essence of consciousness. However, unlike other theories, Orch OR relies on quantum oscillations from microtubules entangled among neurons across the brain, a feature which has yet to be proven, but which may be necessary for cognitive binding and zero-phase lag gamma synchrony. We attempt to strengthen Orch OR with (1) a mathematical Hamiltonian to describe the quantum-classical transition process, and (2) the perspective of complexity theory. We hope to integrate Orch OR with other models spanning many space-time scales.

Orch OR states that phenomena such as the discrete perception of time unfolding, the subjective wholeness of perception, spatio-temporal binding, causal free will (Hameroff, 2012), mechanisms of anesthesia, and the so-called “hard problem" are explainable aspects of consciousness. It organizes these phenomena within a systematic framework capable of explaining possible underlying links and offering interpretations of related philosophical issues (Wiest, 2025).

We believe that, at present, the Orch OR model represents the most comprehensive and bold attempt to propose a framework for the hard problem of consciousness and other features that are connected to it. The foundations of the Orch OR model include Penrose's objective reduction conjecture (Penrose, 1989, 1994, 1996, 2014b,a) and the concept of orchestration in neural processes (Hameroff and Penrose, 1996, 2014; Hameroff, 2001). To our knowledge, there is one computational study that reports explicit theoretical calculations estimating decoherence times and gravitational collapse times in microtubules (Hagan et al., 2002). These coherence times were estimated to range from 10^−6^ to 10^−4^ seconds. Coherence times within this range have also been observed experimentally in microtubules (Saxena et al., 2020), and are considered sufficient for Orch OR processes. We have not found theoretical or computational studies about how, *e.g*., textcolorvioletslower synaptic activity, membrane potentials, calcium ion flows orchestrate the quantum dynamics of the microtubules's lattice. Experimental studies have investigated electronic conduction, excitonic coupling, and electromagnetic resonances in microtubules (Sahu et al., 2013b,a, 2014; Saxena et al., 2020; Kalra et al., 2023). Additional research has explored the possibility of quantum effects in brain structures, including MRI observations of cardiac-evoked zero quantum coherence signals in the brain (Kerskens and érez, 2022), and theoretical models describing coherent excitations in biological systems (Frölich, 1968, 1970, 1975; Pokorný, 1999, 2004). These elements are combined into a framework that is both intellectually engaging and ambitious in its aim to connect gravitation, quantum physics, neuroscience, consciousness, and anesthesia.

We regard this narrative not as a finished structure, but as a foundation, an invitation to contribute with rigorous analysis and an open mind. In this paper, we identify key ideas of the Orch OR model and examine how their internal relations could be analyzed using physically grounded tools. Rather than offering a critique or a generalization, we want to integrate quantum-classical dynamics in the Orch OR framework to describe the evolution of brain structures before and after quantum state reduction. This perspective emphasizes the orchestration process and seeks to formulate it in rigorous physical and mathematical terms, by means of quantum-classical Hamiltonians (Sergi and Kapral, 2003, 2004, 2005; Grimaldi et al., 2021) and the quasi-Lie algebra of quantum-classical brackets (Sergi, 2005; Sergi et al., 2018). In doing so, we want to enlarge the possibilities of the model without altering its foundational premises. For clarity, our specific goal in this work is to propose that the framework of orchestration might find its natural formal expression within a quantum-classical theory (Sergi et al., 2023b, 2025, 2023a, 2018; Sergi, 2015, 2006, 2005; Sergi and Kapral, 2004; Sergi, 2015; Grimaldi et al., 2021). Our paper contributes to the scientific debate on the unfolding of brain dynamics at the boundary between the quantum and classical realms, and aims to help develop Orch OR into a more predictive and testable framework.

This paper is structured as follows. In Section 2, we discuss objective reduction. The second main idea of the Orch OR model, *i.e*., orchestration, is presented in Section 3. In Section 4, we outline a quantum-classical framework that could offer a dynamical description of the classical modulation of quantum events in Orch OR, with the aim of supporting its integration into a testable and predictive multiscale model. Section 5 addresses the quantum dynamics of microtubules. In Section 6, we describe how Professor Hameroff has skillfully used the Orch OR model, specifically the randomization of orchestrated quantum effects in microtubules, to develop a compelling narrative for explaining anesthesia. In Section 7, we discuss how the Orch OR model tackles big questions such as stimulus integration, free will, and the subjective sense of time's irreversibility associated with awareness. In Section 8 we discuss our thoughts about complexity and the meaning of quantum-classical complexity. Finally, in Section 9, we present our main conclusions and final remarks.

## 2 Orch OR, general relativity and quantum gravity

The orthodox interpretation of quantum mechanics considers the quantum state ontologically real (Neumann, 1932). One of its main faults is that it fails to provide a unique and rigorous account of how the classical limit emerges (Wick, 1995; Holland, 1993; Weinberg, 2013). Being a linear theory, it obeys the superposition principle, according to which a system can occupy two quantum states at the same time. If one assigns an ontological status to the quantum states, then the superposition principle does not obey the rule of reality. In the Copenhagen interpretation (Neumann, 1932), only an act of measurement can destroy a superposition and, therefore, create reality as we know it, with systems only occupying a single state at a time. However, since in the Copenhagen interpretation (Neumann, 1932), any act of measurements implies an observer and a measurement, the reality associated with it, is subjective. Typically, the collapse of the wave function is introduced in an *ad hoc* manner to force, although in a probabilistic way, the correspondence with the classical world. This has led to interpretations of quantum mechanics that border on metaphysics, such as the many-worlds interpretation (DeWitt, 1970; DeWitt and Graham, 1973; Deutsch, 1997; Wallace, 2012; Saunders et al., 2010).

Penrose developed the idea that the probabilistic and observer-dependent nature of collapse could be dispensed with by using gravity as a constraint on the lifetime of generic superpositions. This is implemented by considering quantum superpositions not just as superpositions of physical states, but as superpositions of spacetime geometries. Drawing on Einstein's general relativity, where mass and energy curve spacetime, Penrose proposed that a quantum particle in a spatial superposition corresponds to a superposition of distinct spacetime curvatures, differing at the Planck scale. When the gravitational self-energy of this superposition reaches a certain threshold, the state undergoes an objective reduction (OR), spontaneously collapsing to a definite geometry (Penrose, 1996, 2014b,a). The timescale for quantum state reduction is determined by the gravitational self-energy *E*_G_ of the difference between the mass distributions of the superposed states. This leads to the characteristic relation:


(1) $$\begin{array}{l} \tau \sim \frac{\hbar }{E_{\text{G}}}, \end{array}$$

where τ is the mean lifetime of the superposition and ℏ is the reduced Planck's constant. According to Penrose (Penrose, 1996, 2014b,a, 1989, 1994), the exact time of the collapse cannot be predicted by any currently known physical law. Instead, it is hypothesized to follow a yet-to-be-formulated principle emerging from the unresolved conflict between general relativity and quantum mechanics (Penrose, 1989, 1994, 1996, 2014b,a). The law proposed in Equation 1 is said to be neither random nor algorithmically deterministic, but rather deterministic and non-computable.

Objective reduction, with its threshold time given by Equation 1, provides a physical solution to the quantum-to-classical transition (Wick, 1995; Holland, 1993; Weinberg, 2013) which is neither derived from the currently known laws of physics nor computable by any algorithms using a finite number of steps. Reasoning about Gödel's theorem and free will, Penrose concluded that objective reduction could also be the root of what he called proto-consciousness (Penrose, 1989, 1994). In fact, the central assumption of Orch OR is that consciousness arises exclusively through objective reduction (OR). More precisely, Orch OR posits that the collapse of the quantum state of the microtubule lattice within the neuronal cytoplasm constitutes a proto-conscious event. Coherent quantum dynamics entangle the quantum states of individual microtubules, producing a unified quantum state for the entire lattice (Penrose, 1994; Hameroff and Penrose, 1996, 2014; Hameroff, 2001, 2012). In summary, the OR model offers a compelling unification of quantum mechanics and gravity, and gives the Orch OR framework its unique character.

## 3 Orchestration

The Orch OR framework posits that each objective reduction (OR) event corresponds to a discrete moment of subjective experience, or proto-conscious qualia, occurring when quantum superpositions self-collapse into definite classical states. In this view, OR events in isolated systems would give rise to random, disconnected moments, metaphorically described as proto-conscious noise, lacking continuity, memory, or context. These have been likened to the tones of musicians individually tuning their instruments. In contrast, Orch OR proposes that in the brain, OR events are orchestrated across entangled microtubule networks, forming temporally and spatially coherent patterns of reduction. This orchestration is proposed to underlie unified conscious states, resonating and interfering across multiple scales, much like chords and harmonies in a musical composition. The orchestration, realized in part by classical processes occurring in the brain, synchronizes all OR events within individual neurons (Penrose, 1994; Hameroff and Penrose, 1996; Penrose and Hameroff, 2017; Hameroff and Penrose, 2014; Hameroff, 2001). The synchronization of multiple OR events gives rise to conscious experience.

An analogy can be made with a classical system: consider many pendulum clocks, each initially out of phase, but all mounted on the same wall. Because the shared wall transmits vibrations among the clocks, constructive and destructive interference leads them to synchronize over time. Orchestration in Orch OR is a similar process, involving ‘quantum clocks' inside neurons, with the classical processes of the brain acting as the shared “wall." This analogy aligns with more recent interpretations of microtubules as time crystals, structures that may exhibit intrinsic periodicity and support coordinated timing of OR events. (Wilczek, 2012; Zhang et al., 2017; Mi et al., 2022; Sahu et al., 2013b,a, 2014; Saxena et al., 2020).

According to Orch OR (Hameroff and Penrose, 1996; Penrose and Hameroff, 2017; Hameroff and Penrose, 2014), orchestration is caused by processes such as resonance, synaptic activity, membrane dynamics, cytoskeletal regulation [*e.g*., MAPs (Goodson and Jonasson, 2018; Steiner et al., 1990), microtubule-associated proteins, and CAMKII (Waxham, 2009; Baratier et al., 2006; Craddock et al., 2012b; Vallano et al., 1985; Gradin et al., 1997; Schulman et al., 1985)], biochemical activity (*e.g*., calcium waves, GTP hydrolysis), and, *e.g*., anesthesia. These are proposed to modulate or “tune" the quantum superposition within microtubules, without collapsing it. Therefore, orchestration is the quantum coherent dynamics, embedded in the classical environment provided by brain structures, unfolding before an OR event occurs.

## 4 Quantum-classical theory

If the interplay between the coherent dynamics of microtubules and the dynamics of synapses, membranes, cytoskeleton, MAPs (Goodson and Jonasson, 2018; Steiner et al., 1990), calcium waves, GTP *et cetera* invokes both quantum and classical phenomena, then the corresponding modeling requires an advanced formalism that treats quantum and classical degrees of freedom on the same footing (Sergi et al., 2023b, 2025, 2023a, 2018; Sergi, 2015, 2006, 2005; Grimaldi et al., 2021). At the same time, expecting that quantum-classical dynamics does generate quantum transitions, destroying quantum superpositions, is not consistent with what experiments and quantum-classical theory (Sergi et al., 2023b, 2025, 2023a, 2018; Sergi, 2006, 2005; Sergi and Kapral, 2004; Sergi, 2015; Grimaldi et al., 2021) tells us regarding nonadiabatic dynamics. Quantum-classical theory allows for a consistent description of mutual influence and back-reaction between quantum and classical variables, while also enabling the derivation of correlation functions, spectra, and thermodynamic properties that can be compared directly with experimental data. Importantly, this proposal is not in opposition to Orch OR. Rather, it aims to refine and formalize some of its technical aspects, with the goal of enhancing the model's mathematical rigor and predictive power.

It is hard to dispute that orchestration operates within the domain of quantum-classical dynamics, unfolding at the boundary between quantum and classical processes in the brain. The theory of Quantum-classical systems (QCSs) (Balescu, 1975; Zhang and Balescu, 1988; Osborn and Molzahn, 1995; Osborn et al., 1999; Sergi et al., 2023b, 2025, 2023a; Sergi, 2006; Sergi et al., 2018; Sergi, 2005; Sergi and Kapral, 2004; Sergi, 2015; Grimaldi et al., 2021) provides a framework for modeling coupled subsystems with both quantum and classical features. By means of suitable generalizations, this approach can also derive the formal quantum-classical limit of completely quantum correlation functions (Sergi and Kapral, 2004), as well as treat the non-Hermitian dynamics of the quantum subsystem. (Sergi, 2015; Grimaldi et al., 2021). Besides mathematical formalism, there are many efficient algorithms that have proven to be suitable for the simulation of condensed matter systems (Martens and Fang, 1997; Horenko et al., 2002; Wan and Schofield, 2000; Santer et al., 2001; Horenko et al., 2004; Sergi et al., 2003; Sergi and Kapral, 2003, 2005; Sergi and Petruccione, 2010; Uken et al., 2013, 2011; Uken and Sergi, 2015; Sewran et al., 2015; Hanna and Kapral, 2005; Liu et al., 2022).

The theory of QCSs employs Wigner symbols $\tilde{W}\left(R,P,t\right)$ to describe the statistical dynamical properties of a composite system comprising quantum and classical subsystems (Osborn and Molzahn, 1995; Osborn et al., 1999). These symbols depend on quantum variables, such as $\hat{r}$, and classical phase-space coordinates *R* and *P*, representing positions and momenta of, for example, a set of particles. When the Wigner symbol is expanded in a complete basis of the quantum subsystem, it becomes a matrix-valued Wigner function (Zhang and Balescu, 1988; Balescu and Zhang, 1988). The knowledge of a matrix-valued Wigner function allows one to calculate statistical properties by tracing over quantum operators and integrating over classical phase space. Such an approach also allows one to calculate correlation functions and nonequilibrium thermodynamic property (Sergi and Kapral, 2003, 2005).

The theory of QCSs lets one adopt a quantum statistical perspective in which ensemble averages over QCSs dynamical variables, both operators and phase space oordinates, bridge quantum and classical descriptions. Such systems naturally appear in contexts ranging from condensed matter systems (Balescu, 1975; Zhang and Balescu, 1988; Osborn and Molzahn, 1995; Osborn et al., 1999; Sergi et al., 2023b, 2025, 2023a; Sergi, 2005, 2006; Sergi et al., 2018) to quantum processes in classical gravitational fields (Birrell and Davies, 1982; Wald, 1994; Ford, 1982; Kuo and Ford, 1993; Sakharov, 1967; Visser, 2002; Verlinde, 2011; Jacobson, 1995; Oh et al., 2018; Adler, 1982; Boucher and Transchen, 1988; Diósi, 1987, 1989; Penrose, 1996, 2014b,a).

We want now to give a sketch of this theory. We consider a quantum and a classical subsystem, Q and C, respectivily. The classical approximation for C is justified because we are considering those cases in which Λ, *i.e*., the de Broglie wavelenght of C, is much smaller of λ, the de Broglie wavelenght of Q: Λ < < λ. The classical Hamiltonian of C is written as


(2) $$\begin{array}{l} H_{\text{C}}\left(X\right)=\frac{P^{2}}{2M}+V_{\text{C}}\left(R\right), \end{array}$$

where *X* = (*R, P*) is a compact notation for the phase space coordinates. The quantum Hamiltonian of Q is simply denoted by Ĥ_Q_, while the hybrid, coupling Hamiltonian is denoted by ${\tilde{H}}_{\text{QC}}$. The total Hamiltonian ${\tilde{H}}_{\text{T}}$ is


(3) $$\begin{array}{l} {\tilde{H}}_{\text{T}}\left(X\right)={Ĥ}_{\text{Q}}+H_{\text{C}}\left(X\right)+{\tilde{H}}_{\text{QC}}. \end{array}$$

If we denote by ∇ = (∂_*R*_, ∂_*P*_) the phase space gradient, and by **Ω** the symplectic matrix


(4) $$\begin{array}{l} \Omega =\left[\begin{array}{cc} 0 & 1\\ -1 & 0 \end{array}\right]. \end{array}$$

the theory of QCSs (Balescu, 1975; Zhang and Balescu, 1988; Osborn and Molzahn, 1995; Osborn et al., 1999; Sergi et al., 2023b, 2025, 2023a; Sergi, 2005, 2006; Sergi et al., 2018) establishes that the Wigner symbol $\tilde{W}\left(X,t\right)$ (Osborn and Molzahn, 1995; Osborn et al., 1999) obeys the equation of motion


(5) $$\begin{array}{l} \frac{\partial }{\partial t}\tilde{W}\left(X,t\right)=-\frac{i}{\hbar }{\left[\begin{array}{cc} {\tilde{H}}_{\text{QC}} & \tilde{W}\left(X,t\right) \end{array}\right]}^{\text{T}}\Omega \left[\begin{array}{cc} {\tilde{H}}_{\text{QC}} & \tilde{W}\left(X,t\right) \end{array}\right]\\ \;+\frac{1}{2}\underset{kl}{\sum }\left({\tilde{H}}_{\text{SB}}{\overset{⃖}{\nabla }}_{k}\Omega_{kl}{\vec{\nabla }}_{l}\tilde{W}\left(X,t\right)\right)\\ \;-\frac{1}{2}\underset{mn}{\sum }\left(\tilde{W}\left(X,t\right){\overset{⃖}{\nabla }}_{m}\Omega_{mn}{\vec{\nabla }}_{n}{\tilde{H}}_{\text{QC}}\right)\\ \;\equiv -\frac{i}{\hbar }{\left({\tilde{H}}_{\text{QC}},\tilde{W}\left(X,t\right)\right)}_{\text{QC}}, \end{array}$$

The RHS of the final Equation 5 introduces the quantum-classical bracket written in compact form (Sergi, 2005, 2006; Sergi et al., 2018). Equation 5 shows the detailed form of the quantum-classical bracket. The first term in the RHS of Equation 5 is the commutator is the commutator written in matrix form; the last two terms in the RHS of Equation 5 are Poisson brackets (Goldstein, 1980) written in symplectic form.

The quantum-classical bracket is a quasi-Lie bracket (Sergi, 2005, 2006; Sergi et al., 2018) that fails to satisfy the Jacobi identity:


(6) $$\begin{array}{l} {\left({\tilde{\chi }}_{1},{\left({\tilde{\chi }}_{2},{\tilde{\chi }}_{3}\right)}_{\text{QC}}\right)}_{\text{QC}}+\text{cyclic permutations}\neq 0. \end{array}$$

This lack of closure of the quantum-classical algebra reflects the irreversible mixing of quantum features during the system time evolution.

Even with an initially uncorrelated state


(7) $$\begin{array}{l} {\tilde{W}}_{\text{QC}}\left(\tilde{\chi }\right)=W_{\text{C}}\left(X\right){\hat{W}}_{\text{Q}}, \end{array}$$

time propagation


(8) $$\begin{array}{l} \tilde{W}\left(X,t\right)=e^{-\left(i/\hbar \right){\left({\tilde{H}}_{\text{QC}},...\right)}_{\text{QC}}}\tilde{W}\left(X\right) \end{array}$$

inevitably generates correlations even when QC coupling is weak. Our conclusion is that, despite conserving energy and probability (the propagator is unitary), the quantum-classical equation of motion in Equation 5 introduces effective irreversibility by entangling the two subsystems.

The quantum-classical bracket formalism has also been derived as a first-order approximation of the partially Wigner-transformed commutator for fully quantum bipartite systems, see (Sergi et al. 2023a) for a short derivation. It offers a consistent framework to model systems where one component is effectively classical, such as molecular dynamics with classical nuclei and quantum electrons, or quantum particles in a classical gravitational field. In these cases, subsystem energies are not conserved individually, reflecting their open-system character.

Given suitable initial conditions, Equation 5 can be integrated by a number of algorithms (Martens and Fang, 1997; Horenko et al., 2002; Wan and Schofield, 2000; Santer et al., 2001; Horenko et al., 2004; Sergi et al., 2003; Sergi and Kapral, 2003, 2005; Sergi and Petruccione, 2010; Uken et al., 2013, 2011; Uken and Sergi, 2015; Sewran et al., 2015; Hanna and Kapral, 2005; Liu et al., 2022), depending on the system and its defining parameter space. Once $\tilde{W}\left(X,t\right)$ is known, quantum-classical statistical averages can be calculated as


(9) $$\begin{array}{l} \langle \tilde{\chi }\rangle ={\text{Tr}}^{\prime }\int dX\;\tilde{W}\left(X,t\right)\tilde{\chi }\left(X\right), \end{array}$$

while time correlation functions are defined by


(10) $$\begin{array}{l} C_{\chi \xi }\left(t_{2},t_{1}\right)={\text{Tr}}^{\prime }\int dX\;\tilde{\chi }\left(X,t_{2}\right)\tilde{\xi }\left(X,t_{1}\right)\tilde{W}\left(X\right), \end{array}$$

where


(11) $$\begin{array}{l} \tilde{\chi }\left(X,t_{2}\right)=e^{\left(i/\hbar \right){\left({\tilde{H}}_{\text{QC}},...\right)}_{\text{QC}}}\tilde{\chi }\left(X\right), \end{array}$$

and $\tilde{\xi }(X,t_{1})$ defined by the analogous formula to Equation 11.

We believe that the quantum-classical formalism we sketched in this section (Balescu, 1975; Zhang and Balescu, 1988; Osborn and Molzahn, 1995; Osborn et al., 1999; Sergi et al., 2023b, 2025, 2023a; Sergi, 2005, 2006; Sergi et al., 2018; Sergi and Kapral, 2004; Sergi, 2015; Grimaldi et al., 2021), and that has been applied in many works of ours (Sergi et al., 2003; Sergi and Kapral, 2003, 2005; Sergi and Petruccione, 2010; Uken et al., 2013, 2011; Uken and Sergi, 2015; Sewran et al., 2015), including some directly addressing brain dynamics (Sergi et al., 2025, 2023a), could be used to perform computer simulations within the framework of orchestration.

## 5 Microtubules and quantum dynamics

Microtubules are cylindrical polymers formed by the self-assembly of tubulin, one of the most abundant proteins in the brain and a major component of the neuronal cytoskeleton. Each tubulin dimer consists of α- and β-tubulin monomers arranged in a head-to-tail fashion, conferring intrinsic polarity to the polymer. This polarity stems from the dimer's asymmetric structure and directional polymerization (Nogales et al., 1998; Desai and Mitchison, 1997): the plus end, where β-tubulin is exposed, typically elongates more rapidly, whereas the minus end, capped by α-tubulin, is often anchored near the centrosome or within the neuronal soma. In neurons, microtubules exhibit a highly specialized organization distinct from that of other somatic cells. While axonal microtubules are uniformly oriented and continuous, dendrites and the soma contain short, discontinuous segments arranged in mixed-polarity networks (Hameroff and Watt, 1983; Hameroff, 1998; Craddock et al., 2015, 2017; Jibu et al., 1994). The mixed-polarity microtubules are particularly abundant in the somata and apical dendrites of layer 5 pyramidal neurons. This unique neuronal architecture suggests that microtubules may serve roles beyond classical structural support.

In addition to their established involvement in mitosis, axonal growth, intracellular transport, and synaptic plasticity, microtubules have also been implicated in memory mechanisms and theories of consciousness. Of particular relevance to the Orch OR framework is the observation that microtubules support a broad range of vibrational modes and electromagnetic resonances (Sahu et al., 2013b,a, 2014; Saxena et al., 2020; Kalra et al., 2023). Notably, early computational models proposed that the mixed-polarity organization of dendritic microtubules could enable recursive information processing, potentially relevant to conscious experience (Rasmussen et al., 1990). While still speculative, this hypothesis encourages further exploration of the computational functions of cytoskeletal structures in the brain. It has also been proposed that mixed-polarity microtubules can support interference patterns arising from slight frequency differences in their oscillatory behavior (Rasmussen et al., 1990). These interference effects could produce slower beat frequencies in the hertz range, potentially contributing to EEG rhythms (Hameroff and Penrose, 2014). While still speculative, this mechanism suggests a possible link between subcellular microtubule dynamics and large-scale neural activity.

According to Orch OR, a coherent quantum state can form within the hydrophobic regions of individual microtubules (Hameroff and Penrose, 1996; Penrose and Hameroff, 2017), stabilized by benzene/phenyl and indole rings. This state is proposed to involve nonlinear oscillatory dynamics of the highest occupied and lowest unoccupied molecular orbitals (HOMO and LUMO) of these aromatic structures. Quantum synchronization may lead to a unified quantum state encompassing the orbital oscillations across multiple microtubules (Hameroff and Penrose, 1996; Penrose and Hameroff, 2017), with superradiance suggested as a possible mechanism (Babcock et al., 2024). These orbital oscillations are hypothesized to span a broad frequency spectrum, from kilohertz to terahertz, and may contribute to the emergence of conscious experience (Hameroff and Penrose, 1996; Penrose and Hameroff, 2017). In parallel, slower oscillations corresponding to characteristic neural rhythms, alpha, beta, gamma, and theta waves, are seen as macroscopic manifestations of neuronal network activity governed by classical mechanical (Goldstein, 1980), electromagnetic (Jackson, 1998), and thermodynamic [62] principles. These considerations imply that, while Orch OR is rooted in quantum mechanics (Neumann, 1932; Weinberg, 2013), its physical framework calls for a hybrid quantum-classical description (Sergi et al., 2023b, 2025, 2023a, 2018; Sergi, 2015, 2005, 2006; Grimaldi et al., 2021). Microtubules also appear to exhibit coherent oscillations and resonances in self-similar patterns (“triplets-of-triplets”) recurring every three orders of magnitude, from hertz to kilohertz, megahertz, gigahertz, and terahertz, suggesting that they function as polyatomic “time crystals” capable of supporting Orch OR across scales (Saxena et al., 2020).

Quantum optical effects involving the π-electron resonance orbitals of aromatic rings are proposed to initiate quantum processes. Normally, the mass of electrons is negligible compared to nuclei, and their influence is ignored under the Born-Oppenheimer approximation. However, effects involving electron charge, electron-nucleus coupling under electromagnetic fields, or geometric phases in electron clouds may affect nuclear positions. For instance, if an electron undergoes a displacement or superposition on the scale of one nanometer, it could, through electrostatic or structural coupling, displace a carbon nucleus by about one femtometer (*i.e*., the approximate nuclear diameter). Since the Orch OR collapse time τ (see Equation 1) (Penrose, 1996, 2014b,a) is inversely related to the gravitational self-energy *E*_G_, involving more massive systems, such as nuclei, increases *E*_G_ and thus shortens the collapse time.

Three hypothetical scenarios were considered (Hameroff and Penrose, 1996) for estimating *E*_G_ in a single tubulin molecule: (i) partial spatial separation of 10% of its volume; (ii) full spatial separation by the diameter of a carbon nucleus (~1 fm or 10^−15^ m); (iii) full separation by the diameter of a nucleon (*e.g*., a proton or neutron). Among these, scenario (ii) produced the highest *E*_G_ and the fastest collapse. This value was then used to estimate the number of tubulins required to be in superposition to match various neurophysiological timescales via Equation 1. For example, to correspond to a 25-ms gamma-synchrony EEG event, ~2 × 10^10^ tubulins would need to be coherently superposed. Given that a single neuron contains roughly 10^8^-10^9^ tubulins, such a superposition could theoretically involve just a few hundred neurons out of the brain's estimated 10^11^.

However, maintaining coherence for 25 ms is demanding, and premature decoherence may result in only “proto-conscious" effects. By the time of (Hameroff and Penrose 2014), experimental findings from Bandyopadhyay's group (Saxena et al., 2020) had revealed much faster collective oscillations in microtubules, ranging from kilohertz to terahertz. This led to a revised view: if Orch OR events occur at, say, 10 MHz, coherence needs to persist for only 10^−7^ s, a shorter, experimentally supported timescale. Nonetheless, conscious phenomena such as visual gestalts, evoked potentials, and EEG rhythms typically occur at slower frequencies, in the range of hundreds of milliseconds.

A central prediction of the Orch OR model is the emergence of synchronized quantum dipole oscillations in microtubules, in analogy with Fröhlich coherence (Frölich, 1970, 1975). Such oscillations were later discussed in the framework of microtubule dynamics (Hameroff et al., 1998; Rasmussen et al., 1990). Experimental work by Bandyopadhyay's group (Sahu et al., 2013b,a, 2014; Saxena et al., 2020) has reported fractal, scale-free resonant behavior in isolated microtubules, single tubulin proteins, and neurons, including repeating self-similar patterns across several frequency scales (Hz to THz). These observations have been interpreted as suggestive of polyatomic time-crystal-like dynamics, potentially enabling cross-scale coherence and resonant orchestration. Similar ideas were presaged in the work of (Winfree 2001), although without explicit reference to time crystals.

## 6 Orch OR and anesthesia

Anesthesia provides a privileged perspective on consciousness, as it selectively suppresses conscious experience while sparing other brain functions. According to the Meyer–Overton correlation (Meyer, 1937; Overton, 1901), anesthetic potency scales with solubility in non-polar environments, pointing to hydrophobic pockets in neuronal proteins. In the Orch OR framework, such environments are associated with aromatic amino acid residues in tubulin, the fundamental component of microtubules. These regions are hypothesized to support quantum coherence and serve as sites for anesthetic binding. Recent results (Kalra et al., 2023) show anesthetic-induced damping of excitonic propagation in microtubules, consistent with this view and reinforcing the role of tubulin in consciousness-related processes.

Decades of research into anesthetic mechanisms at the level of membrane receptors and ion channels, including GABA_A_ receptors, have not yielded a comprehensive, unified explanation for the loss of consciousness. Notably, (Eger et al. 2008) concluded that no single receptor or combination of receptors could fully account for anesthetic effects. In the same study, they suggest that a “new paradigms” may be needed, one not based on specific molecular targets alone. This perspective opens the door to non-classical targets, such as the quantum-coherent structures proposed in the Orch OR framework.

Molecular and proteomic investigations have highlighted tubulin as a significant target of anesthetic action. (Pan et al. 2006) identified tubulin among a set of ~70 cytoplasmic halothane-binding proteins in mouse/human brain, indicating that half of anesthetic binding occurs beyond membrane sites. Subsequent gene expression studies (Xi et al., 2004; Kalenka et al., 2007) further implicate anesthetics in altering tubulin synthesis.

Remarkably, opto-anesthetic experiments by (Emerson et al. 2013) using a photoactive anthracene-based compound caused immediate anesthesia in transparent tadpoles upon UV illumination. Mass spectrometry analysis confirmed that the compound specifically bound to β-tubulin in microtubules. These results strongly support the case for tubulin as a biologically relevant anesthetic target and reinforce the Orch OR hypothesis linking quantum microtubule dynamics to consciousness (Craddock et al., 2012a).

Recent observations have suggested that microtubule-stabilizing agents, such as paclitaxel and epothilone B, may modulate anesthetic sensitivity in both clinical and preclinical settings (Linganna et al., 2015; Khan et al., 2024). These findings raise the possibility of a cytoskeletal contribution to the mechanisms of anesthesia. Furthermore, computational simulations by (Craddock et al. 2012a) have proposed that anesthetics may dampen quantum van der Waals dipole oscillations among aromatic amino acids in tubulin, correlating with the Meyer-Overton rule. While such models offer an intriguing perspective aligned with quantum theories of consciousness, including the Orch OR framework, these interpretations remain speculative in the absence of direct experimental validation. It is also important to consider that alternative, classical mechanisms, such as alterations in intracellular signaling, membrane receptor dynamics, or neuronal connectivity, may equally account for the observed effects. Therefore, while the role of microtubules in anesthetic action cannot be excluded, further interdisciplinary research is needed to clarify their relevance relative to established membrane-based targets. However the Meyer-Overton correlation points to a single unitary target for all anesthetics, and the only possibility is microtubules.

Recent experimental work (Kalra et al., 2023) has been interpreted as supporting key predictions of the Orch OR model (Hameroff, 2001) by reporting that ultraviolet-induced exciton propagation through microtubules exceeded classical expectations in range and duration, suggesting a quantum optical effect. Moreover, this exciton propagation was reportedly inhibited by clinically relevant anesthetics, including isoflurane and etomidate. While these findings are noteworthy and deserve further investigation, it is important to interpret them with caution. The identification of quantum effects in biological systems at physiological temperature remains technically challenging and open to alternative explanations, such as nonlinear electrodynamical effect (Miller, 2012; Duan et al., 2017; Smirne et al., 2019; Kassal et al., 2013; Briggs and Eisfeld, 2011). In particular, the relation between exciton dynamics and consciousness, as well as the generalizability of such results beyond in vitro systems, remains to be established. We welcome such studies as valuable contributions to the ongoing exploration of consciousness mechanisms but do not regard them as conclusive evidence in favor of any single theoretical framework.

It has been proposed that collective microtubule time crystal behavior enables consciousness to transcend scale (Hameroff and Penrose, 1996; Penrose and Hameroff, 2017; Saxena et al., 2020). Anesthesia acts to block consciousness at the level of terahertz in the microtubules which would otherwise scale up and slow down/interfere with slower visual scenes, conscious epochs, and EEG. Each Orch OR event occurs, selecting conscious perceptions and choices, and classical states of microtubules to enact causal action. Quantum superposition resumes, and isolated quantum events oscillate with classical interludes for output and inputs.

## 7 Orch OR and neuroscience

In addition to conscious moments, quantum effects have been proposed to be necessary for the integration of sensory stimuli (Ghazanfar and Schröeder, 2006; Stein and Stanford, 2008), the spatio-temporal binding of conscious experience (Treisman, 1996; Singer, 2001; Revonsuo, 1999), and the phenomenon of brain-wide zero-phase lag gamma synchrony (Heise et al., 2025; Campo et al., 2019; Viriyopase et al., 2012; Uhlhaas et al., 2009), all of which may raise challenges exceeding the explanatory power of computational models found in the literature (Hameroff, 2022).

In some theoretical frameworks, consciousness and free will are regarded as illusory constructs, *i.e*., as non-active factors (Morsella et al., 2009, 2016). In contrast, other approaches consider them to be interconnected and ontologically real phenomena, emerging either through evolutionary processes (Georgiev, 2024b; Ginsburg and Jablonka, 2019, 2022) or from quantum effects (Stapp, 1993; Jibu and Yasue, 1995; Ho, 2013; D'Ariano and Faggin, 2002; Georgiev, 2025; Fisher, 2015). Orch OR highlights the fact that for consciousness (Georgiev, 2024a; Humphrey, 1983) and free will (Stillman et al., 2011; Baumeister, 2008; Baumeister et al., 2011) to be genuinely real, they must elude algorithmic description (Penrose, 1989, 1994). An automaton, whose thoughts and behaviors are generated solely through computation, would be functionally equivalent to a zombie, fully determined by its programming, and lacking subjective experience or autonomy. This suggests the logical need to invoke a non-computational physical process to account for the existence of real consciousness and free will. In the Orch OR model, building on Penrose's ideas about quantum gravity (Penrose, 1996, 2014b,a), the collapse of the quantum state provides a paradigmatic example of a non-algorithmic process (Neumann, 1932; Wigner, 1961; London and Bauer, 1939; Stapp, 1993). The hypothesis that microtubules might serve as the molecular substrate channeling this quantum state reduction to spatiotemporal scales relevant for consciousness originates from the work of Stuart Hameroff (Craddock et al., 2015, 2017; Jibu et al., 1994).

An important point shared by the Orch OR model (Hameroff, 2012) and the quantum-classical approach (Sergi et al., 2023b, 2025, 2023a; Sergi, 2005, 2006; Sergi et al., 2018; Sergi and Kapral, 2004; Sergi, 2015; Grimaldi et al., 2021) is the breaking of time-reversal symmetry. Whether this asymmetry stems from a deterministic, non-computable law governing quantum state reduction, or from nonadiabatic transitions and interactions with dissipative classical environments, time-irreversible dynamics appears intrinsically linked to the conscious perception of the unidirectional flow of time. As noted by (Smolin 2013), and discussed in our previous work (Sergi et al., 2023b, 2025, 2023a; Sergi, 2005, 2006; Sergi et al., 2018; Sergi and Kapral, 2004; Sergi, 2015; Grimaldi et al., 2021), the breaking of time-reversal symmetry is one of the most immediate and profound features of conscious experience. We all perceive time as flowing in one direction. While unitary quantum dynamics is fundamentally time-symmetric, the introduction of a collapse mechanism modifies this symmetry: past and future are no longer equivalent. In quantum-classical dynamics, a similar asymmetry emerges as an effective description through nonadiabatic transitions.

When quantum-classical equations of motion are written in the adiabatic basis, the matrix-valued Wigner function's dynamics is realized through the propagation of swarms of independent trajectories whose piecewise adiabatic propagation is interspersed by quantum transitions. In such a case, the propagation of the off-diagonal elements of the matrix-valued Wigner function directly provides information about the lifetime of superpositions (coherences). This approach is not in contradiction with the bold ideas proposed by Hameroff and Penrose (Penrose, 1994; Hameroff and Penrose, 1996; Penrose and Hameroff, 2017; Hameroff and Penrose, 2014; Hameroff, 2001, 2012, 2022). In fact, the computer simulation of the nonadiabatic dynamics of the matrix-valued Wigner function requires sampling the times at which the quantum transitions occur. Orch OR, specifically Equation 1, provides a physically motivated law, albeit non-computable, for sampling the times of the quantum jumps, which lead to dephasing. While such a law cannot be implemented directly in quantum-classical simulations, it can be approximated using a stochastic algorithm. Quantum-classical computer models may also be useful to study how the classical environment can modulate the creation of superpositions. In this case, the dynamical contributions to analyze are the transitions from the occupations, *i.e*., the diagonal terms of the matrix-valued Wigner function, to the coherences.

## 8 Quantum-classical complexity

Consciousness is an extraordinarily complex phenomenon (Chalmers, 1995; Nagel, 1974), spanning multiple levels of organization, from physical and biological substrates to cognitive and psychological dimensions (Edelman and Tononi, 2000; Tononi, 2004). Any comprehensive theory must therefore account for this multi-layered nature without neglecting essential aspects such as subjective experience and emotion. It remains an open question whether emotion can exist independently of consciousness (Damasio, 1994; LeDoux, 1996), or whether all affective processes presuppose some degree of conscious awareness. Moreover, consciousness exhibits quantitative variations, from coma to hypervigilance (Laurey, 2005), and qualitative differences, ranging from full self-consciousness (Northoff and Panksepp, 2013) (as in awake humans) to its absence (as in normal dreaming).

These features suggest that consciousness should be approached as a genuine complex system (Dehaene and Changeux., 2011; Werner, 2007; Kelso, 1995; Chialvo, 2010; Bar-Yam, 1993), characterized by hierarchical organization (Werner, 2007), nonlinearity (Kelso, 1995), and emergent behavior (Chialvo, 2010). Typically, the behavior of complex systems (Bar-Yam, 1993) cannot be captured by a single mathematical model; instead, a plurality of irreducible models is required to reflect its many facets. Within the various models that study consciousness, the Orch OR framework occupies a special position, as it addresses the hard problem of consciousness directly. From the perspective of complex systems, quantum-classical systems have two desirable features. The first is that they integrate both quantum mechanics and classical mechanics through a quasi-Lie algebraic structure (Sergi, 2005, 2006; Sergi et al., 2018). This highlights that quantum-classical systems paradigmatically require more than one theoretical model for their description. Second, using the laws of statistical mechanics, they can represent various hierarchical organizations across scales, from the quantum world of atoms and molecules to the macroscopic world of thermodynamics.

This discussion about complex phenomena resisting reduction to a single model, echoes Aristotle's reflections on the problem of definition. Aristotle recognized that not everything admits a precise and univocal definition. Certain concepts are too complex, articulated, or context-dependent to be captured by a single formulation. This view is developed in works such as the *Metaphysics, Topics*, and *Nicomachean Ethics*. In particular, Aristotle emphasized that universal and fundamental concepts often have multiple meanings and, therefore, cannot be explained through a single definition (*Metaphysics* IV, 2, 1003a33). If there is an area in the natural sciences where this epistemic principle is especially evident, it is in the life sciences. Fundamental questions such as “What is life?” (Schrödinger, 2013) or “What is consciousness?” (Chalmers, 1995; Nagel, 1974; Edelman and Tononi, 2000; Tononi, 2004; Damasio, 1994; LeDoux, 1996; Laurey, 2005; Northoff and Panksepp, 2013) resist reduction to a single concept or explanation. This line of reasoning suggests that only multidisciplinary efforts can truly illuminate complex systems.

## 9 Conclusive remarks

The Orchestrated Objective Reduction (Orch OR) model is an intriguing and detailed theory providing a framework for the hard problem of consciousness. It is grounded in the hypothesis that consciousness is non-computable, yet deterministic, and fundamentally linked to the objective reduction of quantum states, a proposal that aims to unify the quantum measurement problem and the hard problem of consciousness within a single physical framework. Orch OR strongly advocates the role of the microtubule lattice as the physical substrate of quantum processes inside neurons. In addition, it proposes orchestration, arising from the dynamics of classical neuronal networks, astrocytes, and other brain structures, modulates these quantum processes. Orch OR also proposes that anesthetics act primarily by damping quantum oscillations (*e.g*., spin transfer, dipole oscillations) within microtubules, while the lattice itself remains polymerized at clinical concentrations. Disassembly may occur only at higher doses (Hameroff and Watt, 1983; Hameroff, 1998; Craddock et al., 2015, 2017; Jibu et al., 1994; Hagan et al., 2002), suggesting that the brain may operate analogously to a quantum computer at the sub-neuronal level (Hameroff, 2022).

The thermally disordered environment of the brain has led some authors to question the feasibility of Orch OR. In particular, (Tegmark 2000) performed calculations on a model of the microtubule environment. His analysis suggested that decoherence would occur extremely rapidly, on timescales ranging from 10^−13^ to 10^−20^ s, effectively making quantum mechanical effects implausible as meaningful contributors to information processing in the brain. (McKemmish et al. 2009) concurred with (Tegmark, 2000) conclusions criticizing the much longer decoherence timescales proposed in (Penrose and Hameroff 2017) and (Hagan et al. 2002). Similarly, (Rosa and Faber 2004) argued that environmental interactions alone are sufficient to account for decoherence in the brain, removing the need for quantum gravitational effects (Penrose, 1996, 2014b,a).

We want to comment on these criticisms. It is important to note that the interior of microtubules is hydrophobic, potentially providing a protective environment that delays decoherence. Acknowledging this, more realistic models of the microtubule interior have yielded revised estimates, extending coherence times by approximately seven orders of magnitude beyond Tegmark's values (Hagan et al., 2002), potentially reaching 10–100 μs. Although still shorter than typical neural timescales (Byrne and Roberts, 2009; Kandel et al., 2012), these megahertz frequency durations may nonetheless permit a meaningful contribution of quantum effects to neural dynamics (Liu et al., 2024).

First-principles Car-Parrinello simulations of the chromophore of the photoactive yellow protein (Sergi et al., 2001) support the notion that structural confinement can suppress decoherence. Additionally, the ability of rigid biomolecular cages to sustain quantum coherence by reducing thermal fluctuations is increasingly recognized in the field of quantum biology (Mukamel, 1997; Prokhorenko et al., 2006). These insights also raise deeper questions about how thermodynamic temperature is defined in nonequilibrium biological systems (Hayashi and ichi Sasa, 2004; Hayashi et al., 2020).

There is also another subtle issue to consider. Decoherence *per se* does not determine the classical limit or the disappearance of quantum effects (Weinberg, 2013). Decoherence makes the density matrix of the system diagonal in a preferred basis (usually the energy basis). However, this still means that the system may have a non-zero probability of occupying different states. Instead, to have a true classical limit, however, systems must occupy single definite states (Weinberg, 2013). This can be achieved only through quantum state collapse, which Penrose's objective reduction guarantees (Penrose, 1989, 1994, 1996, 2014b,a).

Beyond the hard problem, there are many other unanswered questions about consciousness. For instance, the relationship between emotions and conscious awareness is not understood (Damasio, 1994; LeDoux, 1996). Conscious states also vary widely, from coma to hypervigilance (Laurey, 2005), and from full self-awareness to its absence, as in dreaming (Northoff and Panksepp, 2013). These features point to consciousness as a complex system (Dehaene and Changeux., 2011; Werner, 2007; Kelso, 1995; Chialvo, 2010; Bar-Yam, 1993), marked by hierarchy (Werner, 2007), nonlinearity (Kelso, 1995), and emergence (Chialvo, 2010). As with other complex systems (Bar-Yam, 1993), it likely requires multiple mathematical models, each capturing a different and irreducible perspective on the whole.

To address some of these problems, in this paper we propose the application of the quantum-classical theory illustrated in (Sergi et al. 2023b), (Sergi et al. 2025), (Sergi et al. 2023a), (Sergi 2005), (Sergi 2006), (Sergi et al. 2018), (Sergi and Kapral 2004), (Sergi 2015), and (Grimaldi et al. 2021). This approach describes the transition across scales from the quantum level to the classical level of heavy atoms or macromolecules, and further up to thermodynamic behavior and transport phenomena. Conceptually, it is appealing to address consciousness-related phenomena through a theory that unifies quantum and classical descriptions. Quantum-classical theory naturally captures hierarchical, multiscale processes, ranging from the atomic level to thermodynamic regimes, and may therefore provide a suitable model for the layered complexity of brain function. Its predictive power derives from the calculation of quantum-classical averages and correlation functions (Balescu, 1975; Zhang and Balescu, 1988; Osborn and Molzahn, 1995; Osborn et al., 1999; Sergi et al., 2023b, 2025, 2023a; Sergi, 2005, 2006; Sergi et al., 2018; Sergi and Kapral, 2004; Sergi, 2015; Grimaldi et al., 2021; Martens and Fang, 1997; Horenko et al., 2002; Wan and Schofield, 2000; Santer et al., 2001; Horenko et al., 2004; Sergi et al., 2003; Sergi and Kapral, 2003, 2005; Sergi and Petruccione, 2010; Uken et al., 2013, 2011; Uken and Sergi, 2015; Sewran et al., 2015; Hanna and Kapral, 2005; Liu et al., 2022), providing a robust and versatile computational framework.

Our aim is not to introduce an alternative to Orch OR; rather, our approach is intended as a complementary tool. It is a proposal for integrating quantum-classical theory within the Orch OR framework. On one hand, it may provide a numerical strategy for Equation 1 sampling nonadiabatic transition times, which, in quantum-classical dynamics, correspond to losses and revivals of coherence. On the other hand, it may serve to model how classical brain structures modulate quantum states in microtubules and related substrates.

As a next step, we plan to develop numerical models for the computer simulation of ion-channel behavior, axonal electrodynamics, spin dynamics of Posner molecules, and other relevant systems under nonequilibrium conditions. These simulations aim to assess whether quantum features, nonlinear dynamics, chaos, or frustration contribute significantly to the emergence of consciousness.
